# Infection Rate and Drug Resistance of *Ureaplasma urealyticum* and *Mycoplasma hominis* in Patients With Genital Mycoplasma Infection at the National Hospital of Dermatology and Venereology in Vietnam

**DOI:** 10.1155/ijm/4006309

**Published:** 2025-10-09

**Authors:** Luong Huy Vu, Hai Ha Long Le, Hoang Huy Le, Tram Thuy Nguyen, Viet Hoang Nguyen

**Affiliations:** ^1^Department of Laser and Skincare, National Hospital of Dermatology and Venereology, Hanoi, Vietnam; ^2^Department of Dermatology and Venereology, Hanoi Medical University, Hanoi, Vietnam; ^3^Department of Biochemistry, Hematology and Immunology, National Hospital of Dermatology and Venereology, Hanoi, Vietnam; ^4^Department of Clinical Microbiology and Parasitology, Faculty of Medical Technology, Hanoi Medical University, Hanoi, Vietnam; ^5^Department of Bacteriology, National of Hygiene and Epidemiology, Hanoi, Vietnam; ^6^Molecular Pathology Department, Faculty of Medical Technology, Hanoi Medical University, Hanoi, Vietnam

**Keywords:** antimicrobial susceptibility, genital mycoplasma infection, *Mycoplasma hominis*, *Ureaplasma urealyticum*

## Abstract

*Ureaplasma urealyticum* and *Mycoplasma hominis* are significant causes of genitourinary infections, with increasing concerns regarding their antimicrobial resistance (AMR) profiles. This study determined the infection rate and AMR profiles of *U. urealyticum* and *M. hominis* in genitourinary samples from patients at the National Hospital of Dermatology and Venereology, Vietnam. A retrospective analysis of 2207 samples collected from 2018 to 2022 was performed. Isolates were identified and tested for antibiotic resistance using standard methods. Data were analyzed using R software, and statistical associations were assessed using the Cochran–Armitage test. Of the 654 positive cultures, 43.3% were from males and 56.7% from females, with the 25–40 age group most affected (55.7%). *U. urealyticum* was the most prevalent isolate (97.6%), followed by *M. hominis* (28%), with 25.5% showing coinfection. Resistance rates varied significantly for *U. urealyticum*; ciprofloxacin resistance was highest (76%), while josamycin resistance was lowest (6.9%). *M. hominis* showed high resistance to ciprofloxacin (88%), erythromycin (79.2%), and ofloxacin (77%). Coinfections of both species also displayed similarly high resistance patterns. Our study underscores the need for ongoing surveillance of *U. urealyticum* and *M. hominis*. We found that *U. urealyticum* was the predominant pathogen, with resistance patterns differing by species. *M. hominis* exhibited higher resistance overall, while josamycin remained a relatively effective treatment option. Notably, ciprofloxacin resistance was high across all isolates. These findings highlight the urgency and importance of continuously monitoring these pathogens and their resistance profiles.

## 1. Introduction

The facultative anaerobic bacteria *Mycoplasma hominis* (*M. hominis*) and *Ureaplasma urealyticum* (*U. urealyticum*) typically colonize the lower genitourinary tract [1]. These species are major contributors to conditions such as urethritis (excluding gonorrhea), inflammatory conditions of the female reproductive tract, reduced fertility, and other negative pregnancy and neonatal outcomes, despite being widely regarded as commensals. They are also linked to an increased risk of acquiring specific pathogenic illnesses [2–4].

Treatment options for genital mycoplasma infections include tetracyclines (TETs), fluoroquinolones, and macrolides. However, vancomycin and beta-lactam antibiotics are useless against *Ureaplasma* and *Mycoplasma* species because they lack a cell wall. The two species recognized to be harmful in the urogenital tract were traditionally effectively treated with cyclines (doxycycline [DOX], minocycline), josamycin (JOS), and fluoroquinolones [5–7]. In recent years, the misuse of antibiotics, repeated infections, and other factors in clinical health settings and the community have contributed to the increasing drug resistance of *U. urealyticum* and *M. hominis*, posing challenges in achieving standardized clinical treatment.

Epidemiological characteristics of genital infections, including the distribution of bacteria and their antibiotic susceptibility, are known to vary across geographical regions and over time [8]. This variation is likely due to diverse cultural and behavioral factors. Understanding these regional differences is crucial for effective treatment and prevention strategies. While regional variations in the epidemiology of genital mycoplasmas are well established, specific data concerning the prevalence and antimicrobial susceptibility of *U. urealyticum* and *M. hominis* in Vietnam are currently lacking. Thus, this research endeavors to define the prevalence of infection and drug resistance characteristics of *U. urealyticum* and *M. hominis* isolates obtained from genitourinary samples of patients at the National Hospital of Dermatology and Venereology in Vietnam.

## 2. Materials and Methods

### 2.1. Sample Size and Sample Collection

The cross-sectional study included 2207 outpatients who visited the National Hospital of Dermatology and Venereology in Vietnam between 2018 and 2022. Clinical samples were obtained from vaginal discharge, prostatic fluid, semen, first-void urine, and vaginal–cervical fluid by qualified nurses or doctors. As directed by the manufacturer, genital mycoplasmas were detected, and their antibiotic susceptibility was tested in patient samples using a commercial Mycoplasma IST2 assay (M-IST2, bioMérieux, France). Urethral samples were collected from men using cotton swabs, which were gently inserted 1.5–2 cm into the urethra, rotated, and held for 10–15 s to obtain urethral epithelial cells. Similarly, cervicovaginal specimens from women were taken with cotton swabs inserted 2–3 cm into the cervical canal, rotated, and kept for 10–15 s to scrape the endocervical epithelial cells. The cotton swab was withdrawn without touching the vaginal walls. For genital mycoplasma testing, duplicate swabs from a single patient were not included.

All samples were collected into liquid transport medium R1. After 10 s of vortexing swabs in the R1 transport medium, 3 mL of the medium was used to rehydrate the urea–arginine-containing lyophilized selective growth media R2. The presence or absence of genital mycoplasmas was next assessed by adding 50 *μ*L of R2 medium to Mycoplasma IST strip wells during reconstitution and shaking. One or two drops of mineral oil were added to keep each well from drying out. Bacterial growth was assessed after 48 h at 37°C, and color changes were noted at 24 and 48 h. In the culture medium, a color transition from yellow to orange–red, reflecting a pH elevation, is interpreted as evidence of mycoplasma growth, typically suggesting a concentration of greater than > 10^4^ CFU/mL. As directed by the kit, antimicrobial susceptibility was assessed by observing for a red color shift in the test strip as a result of each antimicrobial agent. The manufacturer defined resistance breakpoints for nine antibiotics, expressed in milligram/liter, at two concentrations. Interpretation of susceptibility (*S*), intermediate resistance (*I*), or resistance (*R*) was based on these breakpoints, specifically: DOX—susceptible at concentrations ≤ 4 mg/L and resistant at ≥ 8 mg/L; JOS—*S* ≤ 2 mg/L, *R* ≥ 8 mg/L; ofloxacin (OFX)—*S* ≤ 1 mg/L, *R* ≥ 4 mg/L; erythromycin (ERY)—*S* ≤ 1 mg/L, *R* ≥ 4 mg/L; TET—*S* ≤ 4 mg/L, *R* ≥ 8 mg/L; ciprofloxacin (CIP)—*S* ≤ 1 mg/L, *R* ≥ 2 mg/L; azithromycin (AZM)—*S* ≤ 0.12 mg/L, *R* ≥ 4 mg/L; clarithromycin (CLR)—*S* ≤ 1 mg/L, *R* ≥ 4 mg/L; and pristinamycin (PRI)—susceptible at < 2 mg/L and resistant at ≥ 2 mg/L. In these designations, “*R*” denotes resistant, “*I*” intermediate, and “*S*” susceptible.

### 2.2. Statistical Analysis

Data for the study included *U. urealyticum* and *M. hominis*, patient age, year of *U. urealyticum* and *M. hominis* isolation, and antimicrobial susceptibility testing. Data were analyzed using R Software Version 4.3, and statistical calculations were applied to assess any association between the variables studied. Cochran–Armitage's test calculated the *p* trend and *Z* statistics. A significance level of *p* < 0.05 was established for this study.

To identify independent factors associated with antimicrobial resistance, multivariable logistic regression analysis was performed for each antibiotic. The outcome variable for each model was the resistance (resistant vs. susceptible/intermediate) to a specific antibiotic. Independent variables included in the models were sex (male vs. female), age (continuous), year of isolation (2018 as reference), STD diagnosis (yes vs. no), and the presence of *U. urealyticum* (positive vs. negative) or *M. hominis* (positive vs. negative). Adjusted odds ratios (aORs) and their corresponding 95% confidence intervals (CIs) were calculated. To enhance interpretation and reveal patterns from the multivariable analysis, results were visualized using a forest plot. A two-sided *p* value of < 0.05 was established as the threshold for statistical significance for all analyses. All statistical analyses were conducted using appropriate functions from standard R packages, including stats for logistic regression and ggplot2 for visualization.

## 3. Results

### 3.1. Prevalence of Genital Mycoplasma Infection

A total of 2207 clinical samples collected from outpatients were analyzed in the present study from 2018 to 2022, with an overall prevalence of genital mycoplasma infection of 29.6% (654/2.207). Women accounted for more positive cases than men, at 56.7% (371/654) compared to 43.3% (283/654). Based on age distribution, 169 patients (25.8%) were under 25 years old, 364 patients (55.7%) were between 25 and 40 years old, and 121 patients (18.5%) were over 40 years old (Table 1).

**Figure 1:**
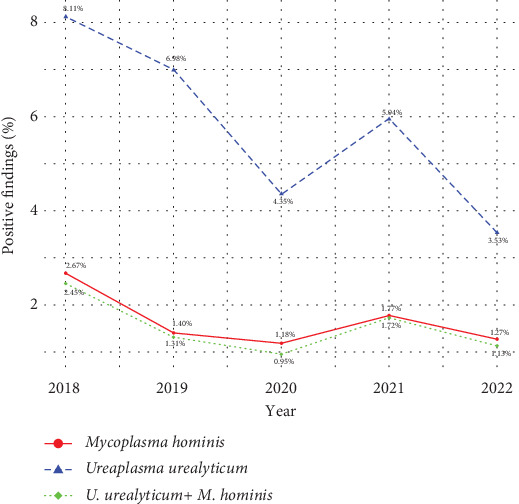
Trend in the prevalence of *Ureaplasma urealyticum* and *Mycoplasma hominis.*

Of the 654 samples with a positive finding, *U. urealyticum* infection (638, 97.6%) was much more prevalent than *M. hominis* (183, 28%). Trends in the prevalence of genital mycoplasma between 2018 and 2022 are shown in Figure 1. In general, positive samples of *M. hominis* were found to be low (ranging from 1.18% to 2.67%) during the 5-year period of study, whereas the positive samples of *U. urealyticum* ranged from 3.53% to 8.11%.

### 3.2. Antimicrobial Susceptibility Testing

The result of the antibiogram of *U. urealyticum* and *M. hominis* isolates to different antimicrobial agents, according to the year of isolation, is presented in Tables 2 and 3. It was noted that the resistance pattern of *U. urealyticum* isolates showed a significant upward trend, particularly in resistance to AZM, increasing from 17.3% to 28.2% (*z* = 2.4305, *p* = 0.0151). No statistically significant changes were observed in the antimicrobial susceptibilities of *M. hominis* isolates during the analyzed period.

**Figure 2:**
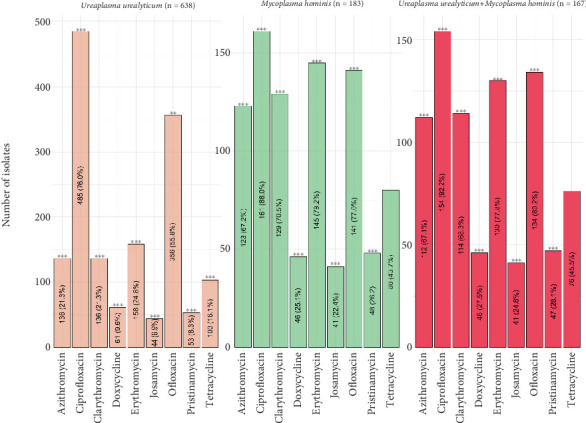
Resistance of genital mycoplasmas to antimicrobial agents assessed in the study. ⁣^∗^*p* < 0.05; ⁣^∗∗^*p* < 0.01; ⁣^∗∗∗^*p* < 0.001.

The antibiograms of *U. urealyticum* and *M. hominis* and coinfections of both species to several antimicrobial agents are depicted in Figure 2. Each isolate exhibited resistance to a minimum of one antimicrobial agent. *M. hominis* isolates showed a higher degree of resistance to many drugs. *M. hominis* isolates were 88.0% resistant to CIP, followed by 79.2% resistant to ERY, 77.0% resistant to OFX, 70.5% resistant to CLR, 67.2% resistant to AZM, 43.7% resistant to TET, 26.2% resistant to PRI, and 25.1% resistant to DOX, and JOS (22.4%) showed the least resistance.

In the study, 485 (76.0%) and 356 (55.8%) of the 638 *U. urealyticum* isolates were resistant to CIP and OFX, respectively (Figure 2). Moreover, 136 (21.3%), 136 (21.3%), and 158 (24.8%) of the 638 *U. urealyticum* isolates showed resistance to AZM, CLR, and ERY, respectively, of the nine antimicrobials assessed. Low resistance was observed for either of the two TETs (DOX and TET) for any *U. urealyticum* isolate, and 6.9% of these isolates were resistant to the macrolide JOS.

In Figure 2, the three antibiotics (DOX, TET, and JOS) were also ineffective against 27.5%–45.5% of the bacteria detected from patients simultaneously infected with *U. urealyticum and M. hominis*. While 75.0%–79.0% of the isolates of *U. urealyticum* were responsive to the three macrolide antibiotics (ERY, CLR, and AZM), over 60% of the isolates of *M. hominis* and isolates from coinfections were resistant to them. Furthermore, two quinolone medicines (OFX and CIP) were ineffective against over 50% of isolates recovered from the research population. Significantly high resistance levels to AZM, CIP, CLR, DOX, ERY, JOS, and PRI (*p* < 0.0001) were seen.

**Figure 3:**
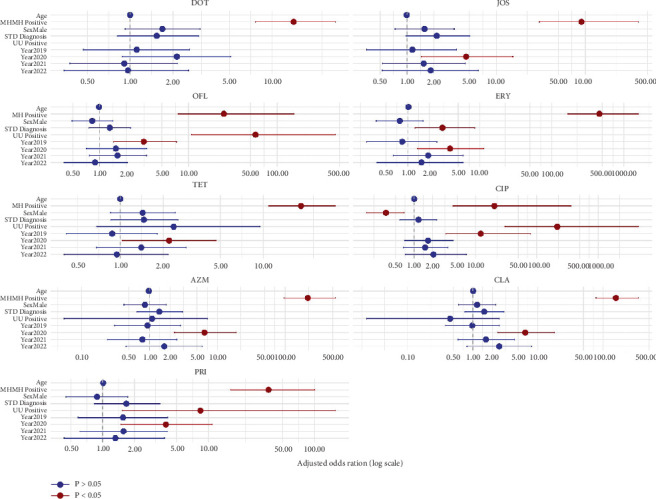
Multivariable logistic regression analysis of factors associated with antimicrobial resistance in *Ureaplasma urealyticum* and *Mycoplasma hominis.*

Multivariable logistic regression analysis identified several independent predictors of antibiotic resistance (Figure 3 and Table S1). Notably, *M. hominis* positivity was the strongest and most consistent factor associated with resistance across nearly all antibiotics, with aORs ranging from 14.1 (95% CI: 7.63–27.7) for DOX to 456 (95% CI: 167–1590) for ERY. Additionally, the year 2020 was significantly associated with increased resistance to several antibiotics, including AZM (aOR = 6.44, 95% CI: 2.35–18.9), CLR (aOR = 6.42, 95% CI: 2.42–17.8), TET (aOR = 2.20, 95% CI: 1.04–4.67), JOS (aOR = 4.60, 95% CI: 1.45–15.4), and PRI (aOR = 3.96, 95% CI: 1.49–10.8), indicating a possible temporal shift in antimicrobial resistance patterns. Year 2019 was also associated with increased resistance to CIP (aOR = 12.1, 95% CI: 3.29–79.1) and OFX (aOR = 3.14, 95% CI: 1.44–7.34). Furthermore, *U. urealyticum* positivity was associated with OFX (aOR = 57.0, 95% CI: 10.8–449) and with CIP (aOR = 216, 95% CI: 30.2–4509). In contrast, male sex was associated with a lower risk of resistance to CIP (aOR = 0.35, 95% CI: 0.17–0.69). Clinical diagnosis of sexual infection (STD) was significantly associated with ERY resistance (aOR = 3.03, 95% CI: 1.26–8.49).

## 4. Discussion

Genital mycoplasmas are frequently isolated from symptomatic patients, and our study was aimed at evaluating the infection rate and AMR profiles of *U. urealyticum* and *M. hominis*. Although the prevalence of *U. urealyticum* and *M. hominis* among patients with genitourinary tract infections varies [8], our findings align with a general understanding of these infections. We found that *U. urealyticum* infections in our study (97.6%) were single infections, whereas *M. hominis* infections were found in 28.0% of patients. The detection rate of coinfections with *U. urealyticum* and *M. hominis* was 25.5%. These findings align with previous reports, and systematic reviews have shown variability in the reported colonization rate of *M. hominis* [9]. While previous studies have estimated *U. urealyticum* prevalence to be higher [10, 11], our findings are consistent with the general understanding that prevalence rates for both organisms can vary across different populations. However, Song et al. observed a lower prevalence for *Ureaplasma* species (31.3%) and *M. hominis* (0.8%) [12]. Although the data provide important insight into local prevalence, they may not reflect the epidemiological situation in other regions, as this study was conducted at a single tertiary care hospital. Differences in antibiotic prescribing practices, patient demographics, and diagnostic approaches across healthcare settings may influence both the detection and resistance rates of *U. urealyticum* and *M. hominis*. Therefore, caution should be exercised when extrapolating these findings beyond the study site. Multicenter or population-based studies are needed to validate and extend these observations and assess the impact of these variables on prevalence estimates in further studies.

Consistent with previous research, our study revealed a higher prevalence of *U. urealyticum* and *M. hominis* in female patients than males. This observation, also demonstrated by Kaprzykowska et al., suggests that women's physiology may predispose them to these infections [13]. The underlying reasons are likely multifactorial, encompassing biological factors such as variations in the urogenital microbiota, vaginal pH, and hormonal fluctuations, which could create a more favorable environment for colonization [14, 15]. Additionally, behavioral factors, including healthcare-seeking behavior and increased likelihood of routine gynecological examinations, may contribute to higher detection rates in women. Numerous factors, including the menstrual cycle, pregnancy, infertility, spontaneous abortion, vaginal contraceptive use, and bacterial infections, are etiologically linked to the prevalence of these organisms [4].

Our study also found a correlation between mycoplasma infections and age. While the highest positive rates in our study were observed in the 25–40 age group (55.7%), this may be related to factors such as being of childbearing age and sexual activity, recognized to be associated with urogenital infections. Indeed, Bayraktar et al. focused specifically on pregnant women, a key group within childbearing age, and demonstrated the prevalence of *U. urealyticum* and *M. hominis* in this population [16], further highlighting the importance of age and related factors in the epidemiology of these infections. In contrast, infection rates significantly declined in individuals over 40 (18.5%). This trend is consistent with epidemiological studies from Hungary, Italy, China, and Korea [17–20]. The varying infection rates across age groups in our study, consistent with observations in other epidemiological studies [21, 22], indicate that age is one factor influencing mycoplasma infection prevalence. However, significant gaps remain in our understanding of the precise causality of these infections and the underlying mechanisms driving age and gender-related differences. Additionally, our knowledge about *Mycoplasma* and *Ureaplasma* species in the urogenital tract is incomplete. These factors highlight the need for further studies in the future.

Over time, the growing resistance of *U. urealyticum* and *M. hominis* to existing antibiotics is worrisome. All the *U. urealyticum* and *M. hominis* isolates resisted at least one antibiotic. The majority of *U. urealyticum* were most resistant to CIP (76.0%), followed by OFX (55.8%) and ERY (24.8%), but were most susceptible to JOS (6.9%) and DOX (9.6%), respectively. Comparable resistant results were noted for CIP and OFX among *U. urealyticum* isolates in Turkey (92.6% and 85.2%, respectively) or in Serbia (83.8% and 48.6%, respectively) [16, 23]. This finding, along with recent reports on the antimicrobial susceptibility of other pathogens such as methicillin-resistant *Staphylococcus aureus* in Vietnam, highlights the urgent need for antibiotic surveillance and effective antibiotic stewardship programs [24]. In contrast, in Germany, Dumke found fewer fluoroquinolone-resistant *Ureaplasma* isolates (7.1%) in comparison with our study [25].

Fluoroquinolones, TETs, and macrolides are commonly used to treat *Ureaplasma* and *Mycoplasma* infections [2]. While DOX and JOS have historically shown good efficacy, our study highlights a concerning trend of rising resistance to fluoroquinolones and macrolides, particularly in *M. hominis* single infections and *U. urealyticum, M. hominis* coinfections, both of which demonstrated higher resistance rates compared to *U. urealyticum* single infections. This rising resistance, consistent with recent reports of increasing AMR in these pathogens, may be attributable to differing drug resistance mechanisms in coinfections and poses a significant challenge to effective treatment. The increased resistance to frequently used antibiotics underscores the critical need for ongoing surveillance and exploring alternative therapeutic strategies.

The multivariable logistic regression analysis revealed important microbiological and temporal antimicrobial resistance predictors in patients with mycoplasma genital infection. *M. hominis* positivity became the strongest and most consistent factor, independently associated with a significant increase in resistance in almost all antibiotics studied. This suggests that *M. hominis* can have or acquire intrinsic resistance mechanisms that warrant closer clinical and microbiological attention. Furthermore, in 2020, several antibiotic resistances to AZM, CLR, TET, JOS, and PRI, among others, were significant, suggesting a possible change in resistance during this period. These findings align with regional and global data indicating a marked increase in resistance during 2020. A Greek survey of nearly 3400 isolates reported a rise in *M. hominis* AZM resistance from ~52.8% in 2017 to ~97.2% in 2022, and CLR resistance surged to nearly 98.5% by 2021 [26]. Similarly, South Korean data showed increasing resistance to PRI among genital mycoplasmas during the same period [27]. Global meta-analysis of *Mycoplasma genitalium* further revealed macrolide resistance increasing from 25.6% (2003–2015) to 56.6% in 2020–2023 [28]. Also, these changes can reflect changes in the use of antimicrobials, diagnostic practices, or broader public health disruptions (such as the COVID-19 pandemic) that have unintentionally influenced resistance trends. We also observed that the positivity of *U. urealyticum* predicted resistance to fluoroquinolones such as OFX and CIP, highlighting species-specific differences in drug sensitivity. Interestingly, male sex was associated with a reduction in the odds of resistance to CIP, indicating potential behavioral, biological, or therapeutic access factors that should be further investigated. In addition, clinical diagnosis of sexually transmitted infections (STDs) may reflect the treatment-driven selective pressures of symptoms of patients and be associated with increased resistance to ERY. Overall, these results highlight the importance of species identification, temporal surveillance, and patient-level factors in understanding and responding to antimicrobial resistance to microbial infections. Integrating such insights into clinical practice can improve the accuracy of empirical therapy and improve the results of treatment.

The increasing prevalence of drug-resistant pathogens poses a significant danger to our capacity to effectively manage infections. Therefore, implementing an antibiotic stewardship program in healthcare facilities is crucial to curb antibiotic misuse. Such a program should designate responsible personnel for prescribing critical antibiotics, ensuring that these valuable antimicrobials are reserved for serious and clinically justified situations.

While a key strength of our study lies in its large sample size, it is important to acknowledge several limitations. First, this retrospective study exclusively focused on symptomatic patients presenting at a specialized dermatology and venereology hospital due to existing genitourinary symptoms or specific clinical indications. Therefore, our findings directly reflect the antimicrobial resistance profiles relevant to active infections within a symptomatic patient cohort. This specificity means our results may not be generalizable to asymptomatic carriers, and the potential for overestimating clinically relevant resistance due to asymptomatic colonization is not applicable in our study context. Second, we relied solely on a commercial phenotypic susceptibility test without precise genotypic confirmation. Although the test is widely used and clinically relevant, the absence of molecular data on resistance mechanisms limits mechanistic interpretation. Third, the absence of precise minimum inhibitory concentration (MIC) values, which, while sufficient for classification, precludes a more granular analysis of resistance levels, especially for isolates near interpretive breakpoints. Fourth, our study did not include detailed clinical background data on patients, such as underlying comorbidities, prior antibiotic use, or immune status factors known to influence antimicrobial resistance. These clinical variables can affect host susceptibility, pathogen persistence, and prior exposure to antimicrobials, potentially introducing variability in resistance patterns. The absence of this information limits our ability to interpret resistance findings in the context of individual patient risk profiles. In addition, the lack of a more in-depth investigation into risk factors associated with urogenital tract infections in our hospital setting would provide valuable insights for implementing targeted interventions. Due to the changing drug resistance spectrum, periodic monitoring of sensitivity testing is significantly important to guide clinical treatment. Regular surveillance of urogenital infections, including monitoring antibiograms of *U. urealyticum* and *M. hominis* and the formulation of definitive antibiotic policies, can help in the early cure of patients, reduce the incidence of genital mycoplasma infections, and prevent the occurrence of resistant trends. The study is, therefore, an opening to facilitate epidemiological studies in the future of Vietnam.

## 5. Conclusions

In summary, this study demonstrated a high prevalence of single *U. urealyticum* isolates in the National Hospital of Dermatology and Venereology setting, posing significant challenges for infection control. Women and groups of childbearing age (25–40 years old) accounted for a higher proportion of positive cases than men. Notably, antibiotic resistance patterns were more prevalent in *U. urealyticum* and *M. hominis* mixed infections and single *M. hominis* infections. CIP and OFX showed low effectiveness, while DOX and JOS remained viable options for urogenital treatment. Due to the emergence of drug resistance being a growing concern, continued monitoring of antibiotic susceptibility patterns and better management of the agent prescriptions in patients presenting with urogenital infections is crucial to preserving the effectiveness of existing treatments.

## Figures and Tables

**Table 1:** Demographics of mycoplasmas isolated from patients of the National Hospital of Dermatology and Venereology.

	**Number of isolates**	**Percentage (%)**
Gender		
Males	283	43.3
Females	371	56.7
Total	654	100
Pathogen		
*U. urealyticum*	638	97.6
*M. hominis*	183	28.0
*U. urealyticum* + *M. hominis*	167	25.5
Total	654	100
Age group		
< 25	169	25.8
25–40	364	55.7
> 40	121	18.5
Total	654	100

**Table 2:** Resistance rate to antimicrobial agents of *U. urealyticum*.

	**2018** **n** = 179	**2019** **n** = 154	**2020** **n** = 96	**2021** **n** = 131	**2022** **n** = 78	**Z**	**p** **trend**
Tetracyclines							
Doxycycline	8.90%	7.80%	13.50%	9.20%	10.30%	0.5337	0.5935
Tetracycline	15.60%	11.00%	20.80%	18.30%	17.90%	1.1858	0.2357
Macrolides							
Azithromycin⁣^∗^	17.30%	15.60%	31.20%	22.10%	28.20%	2.4305	0.0151
Clarithromycin	20.10%	14.90%	30.20%	20.60%	26.90%	1.5268	0.1268
Erythromycin	24.00%	18.80%	29.20%	26.00%	30.80%	1.4987	0.1340
Josamycin	5.00%	4.50%	10.40%	8.40%	9.00%	1.7435	0.0812
Fluroquinolones							
Ofloxacin	55.30%	55.80%	56.20%	58.00%	52.60%	−0.0259	0.9794
Ciprofloxacin	71.50%	79.20%	75.00%	82.40%	70.50%	0.7363	0.4615
Streptogramin							
Pristinamycin	6.70%	6.50%	13.50%	9.20%	7.70%	0.8966	0.3699

*Note: n*: total tested strains, *p* trend and *Z* were calculated by the Cochran–Armitage test.

⁣^∗^Upward trend (*Z* > 0; *p* < 0.05).

**Table 3:** Resistance rate to antimicrobial agents of *M. hominis.*

	**2018** **n** = 59	**2019** **n** = 31	**2020** **n** = 26	**2021** **n** = 39	**2022** **n** = 28	**Z**	**p** **trend**
Tetracyclines							
Doxycycline	18.60%	32.30%	34.60%	23.10%	25.00%	0.5258	0.5990
Tetracycline	35.60%	45.20%	61.50%	43.60%	42.90%	0.8653	0.3869
Macrolides							
Azithromycin	54.20%	80.60%	80.80%	64.10%	71.40%	1.3062	0.1915
Clarithromycin	61.00%	77.40%	92.30%	61.50%	75.00%	0.8820	0.3778
Erythromycin	72.90%	87.10%	92.30%	71.80%	82.10%	0.4656	0.6415
Josamycin	15.30%	22.60%	30.80%	25.60%	25.00%	1.3275	0.1844
Fluroquinolones							
Ofloxacin	81.40%	71.00%	76.90%	74.40%	78.60%	−0.4025	0.6874
Ciprofloxacin	86.40%	87.10%	80.80%	94.90%	89.30%	0.8447	0.3983
Streptogramin							
Pristinamycin	20.30%	29.00%	34.60%	28.20%	25.00%	0.6988	0.4847

*Note: n*: total tested strains, *p* trend and *Z* were calculated by the Cochran–Armitage test.

## Data Availability

The datasets generated and analyzed during the current study are not publicly available due to patient privacy concerns. Access to anonymized data may be requested from the corresponding author, subject to approval by the National Hospital of Dermatology and Venereology's ethics committee.

## Supporting Information

Supporting Information Additional supporting information can be found online in the Supporting Information section. Table S1: Detailed results of multivariable logistic regression analysis of factors associated with antimicrobial resistance in *Ureaplasma urealyticum* and *Mycoplasma hominis.*
